# Indents

**Published:** 1916-07

**Authors:** 


### INDENTS

☞ **Brief Articles of Technical or Scientific Value will receive notice and comment. Contributions invited.**

*Clasps.* For a partial lower rubber denture the best in all respects is the double loop wire clasp, using 18k. 18 gauge gold wire, about one and a half inches. With flat-nosed pliers bend double, leaving one-sixteenth inch space between. Fit with pliers the loop to the buccal side of plaster tooth. It is not necessary that it touch every bit of the surface. As the rubber forms the lingual surface of the clasp, the ends of the wire are turned at right angles to be vulcanized into the rubber. After arranging the teeth and waxing up, leaving sufficient room for the clasp, take it in the pliers warm and drop into place. This clasp is quickly made, easily adjusted and cleaner than the band clasp.—L. P. Haskell in *Review.*

*Enlarging Constricted Root Canals in Upper Bicuspids.* When a constriction in a pulp canal is met with in an upper bicuspid, the mouth lamp can be placed on the lingual or buccal side, and the canal illuminated. The continuation of the canal is seen as a small, black spot. A fine drill in the engine is placed on this spot, and a few revolutions of the engine will cause it to jump through into the canal beyond the constriction.—E. S. Best, *Dental Review.*

*Ether as an Antidote for Cocain and Novocain Poisoning.* Engstad, following the publication of an article in the *Journal of the American Medical Association* in 1910, under the title of "Ether an Antidote of Cocain and Novocain," gives the results of his tests of the action of ether in a large number of cases of poisoning from the alkaloid of cocoa leaves. Case reports from other sources tend to confirm his claims for ether as a safe, and, in the majority of cases, certain antidote for the subtle and dangerous alkaloid cocain, as well as for any of the synthetic preparations. Cocain lowers the blood pressure with a tendency to inhibit the complete systolic action of the heart, especially of the right side. It also inhibits the respiratory action, due to poisoning of the respiratory centers of the brain, the heart becomes flabby, and there may be over-stimulation of the accelerator nerves. Ether, on the other hand, is a rapidly diffusible cardiac stimulant; it stimulates the vasomotor as well as the respiratory centers. It increases the blood pressure and has apparently a special affinity for the toxic elements of the diffusible alkaloids. Engstad used to employ strychnin and morphin in combination for the resuscitation of patients suffering from cocain poisoning, but found it necessary that a remedy should be discovered which could be administered at any time and be instantaneous in its action. Ether is administered as ordinarily given to produce surgical narcosis—that is, to the stage of stimulation of the vasomotor system and of the respiratory centers of the brain and of the pneumogastric nerve, which increases the pulmonary circulation in the first stages. The marked mental excitement is allayed as the patient goes under the influence of the ether, and the effect of the poison rapidly disappears. The individual regains consciousness as soon as the effect of the small amount of ether has disappeared, if its administration is carried only to the stage of mild analgesia. A mask is employed and the ether is given by the drop method. The safe and efficient antidotal action of ether in experimentally produced novocain poisoning in animals has been tested by Dr. Corbit of the University of Minnesota, for the purpose of preventing possible deaths from novocain poisoning.—J. E. Engstad in *Cosmos,* June, 1916.

*Root Filling.* Chloro-percha is introduced into the canal in a consistency proportionate to the size of the foramenal opening; thin in normal adult cases and thicker as the foramen is known to be larger. Enough of this is forced in with canal probes to be sure that it has been carried to or very near to the apical opening. The tip end of a cone, if the canal is fairly large, or a part of one of the "special" round or wire-like gutta-percha points if the canal is very fine, is then introduced and carefully pressed to the end. This distance should be accurately known, being calculated with the aid of the radiograph, which shows the shadow of the wire in the canal. If there is any doubt about this sealing of the end, a radiograph should be taken and developed while the patient is still in the chair.

When the operator feels assured that he has sealed the end, he then proceeds with his root filling. Cones or points are introduced one after the other and packed more and more forcibly. At first the gutta-percha is readily softened by the chloroform in the canal, but as the work proceeds what was at first perhaps of the consistency of milk becomes as thick as cream, and then more like cheese; and as more and more gutta-percha is forced in, it grows denser and denser, until at the end a broad-faced plugger may be pressed against it and a mallet used with considerable force for further and final condensation.

Such a gutta-percha canal filling can not shrink through the evaporation of chloroform, because all of the original chloroform has been utilized in the absorption of the added pieces of chloro-percha. Moreover, such a filling is not conical in shape, but assumes the exact form of the canal, however irregular or tortuous it may be, and is even forced into those multiple foramina that we are so frequently warned against.—Dr. Ottolengui in *Dental Items of Interest.*

*The Function of the Saliva.* In a paper in the September issue of the *Biochemical Journal,* Mr. L. A. Maxwell, writing from the physiological laboratory of the Melbourne University, suggests that colloidal starch solutions might absorb pepsin in the same way as charcoal and other powders, and hence inhibit the activity of this enzyme. On the other hand, saliva by hydrolyzing starch might prevent this absorption of pepsin by the colloidal carbohydrate, serving thus as a material aid to digestion. Mr. Maxwell's experiments support this view. He finds that peptic digestion is delayed in the presence of colloidal starch solutions, through absorption of the enzyme. There is a stage in the disruption of the starch molecule at which the absorption of pepsin is lost, this occurring between the amylodextrin and erythrodextrin stage. Unboiled starch does not hinder the action of pepsin, which is a significant fact in connection with the dietetics of herbivora. Further, cooked farinaceous foods—as rice, potato, bread, porridge—inhibit peptic digestion if not first subjected to salivary digestion. It seems clear, therefore, that ptyalin in human saliva plays a considerable part in aiding gastric digestion by hydrolyzing colloidal starch, which otherwise would absorb pepsin. These observations are of great interest, as they may likely enough throw light on the value of active malt extracts and malted foods in dietetics, and particularly in the feeding of infants. It certainly seems an odd physiological fact, as Mr. Maxwell points out, that an enzyme should be secreted in the mouth only to be presently destroyed in the stomach; while, again, a more efficient diastase is present in the secretion of the pancreas. The experiments, too, would appear to afford further evidence of the undesirability of unaltered starch being present in the infant's diet.—*Lancet,* per *Dental Record.*

*Immunity from and Susceptibility to Dental Caries.*

The predisposition of teeth to caries varies in different individuals, and, therefore, so-called "hard" and "soft" teeth do exist. Whether an individual has "hard" or "soft" teeth depends upon the health of the person. Local immunity to caries may be explained as follows: The development of crowns of the permanent teeth extends from the first to the ninth year (not counting the third molar). If at any time disturbances of any of the ductless glands occur, the teeth developed at that time will not be properly calcified and will be disposed to caries. When health is restored, the other teeth develop normally and are immune to caries. According to the state of our present knowledge, the ductless glands exert the greatest influence upon size, development and calcification of the teeth and bones. Pathological changes, or the removal of any of the above-mentioned glands, cause a diminution in the amount of calcium salts deposited in the bones and teeth. If the surgical removal of these glands causes such an ill effect upon the teeth, surely means will finally be found to stimulate the glands in question, so as to perfect the calcification in cases of so-called "soft" teeth.—C. F. Bödecker, *Dental Review.*

*Cleanliness in Synthetic Operations.* To get the best results from synthetic fillings, care must be exercised to insure absolute cleanliness of the mixing slab, spatula and instruments. Of course, these are always washed immediately after they are used, but are they always cleaned with alcohol just before using? They should be in every instance to free them from any foreign substance, which, if present, even in minute quantity, is certain to become incorporated with the mix, and, therefore contaminate it, with the result of an operation not altogether successful. Failure with synthetic as regards color may almost invariably be traced to failure to give due attention to this important detail.

For this purpose I use an inexpensive perfume atomizer or perfumer, as it is called, made by the De Villbis Manufacturing Company, and it is extremely convenient and efficient.

By holding the slab and instruments in front of the atomizer containing alcohol and compressing the bulb only once or twice, you will find the whole surface covered with a fine spray of alcohol, which, when wiped off with a clean dental napkin, removes the last trace of foreign material and leaves the slab, instruments, etc., absolutely clean.

Another excellent use for the alcohol thus atomized is for the cleansing of eye glasses. A great part of humanity use glasses, and I know a large per cent. have trouble in keeping them clear and clean, much to their discomfort, if not actual injury. Glasses cleaned in this manner are perfectly clear and are much appreciated by tired eyes, particularly at the end of the day.—J. W. O'Connell in *Dental Quarterly*.

*Oral Hygiene in the Chesterfield Letters.* Members of the dental profession can but but be interested at the comment made on their work nearly or quite one hundred and fifty years ago by that brilliant wit, Lord Chesterfield. While Chesterfield had ambition to shine as an orator and man of letters, he would probably have been completely forgotten except for the accident in having had preserved, and later published, without any thought on his part that such course would follow, the confidential letters which he wrote to his son. A recent re-reading shows them to be human to a degree. Chesterfield's son was evidently like many sons of the present, and grew into "one of those ordinary men of the world of whom it sufficeth to say that there is nothing to be said."

This son was early sent to Westminster School, where he formed careless habits in dress and personal appearance which were long a trial to his father, and the anxiety with which the father urged the importance of a pleasing personal appearance is a striking evidence of what Lord Chesterfield considered important. In one of his letters he says:

"The best authors are always the severest critics of their own works; they revise, correct, file and polish them, till they think they have brought them to perfection. Considering you as my work, I do not look upon myself as a bad author, and am, therefore, a severe critic. I examine narrowly into the least inaccuracy or inelegancy, in order to correct, not expose them, and that the work may be perfect at last."

Several times in this correspondence do we find reference to the care of the teeth. In 1747 the father writes:

"Do you take care to keep your teeth very clean, by washing them constantly every morning and after every meal? This is very necessary, both to preserve your teeth a great while and to save you a great deal of pain. Mine have plagued me long and are now falling out, merely for want of care when I was your age. Do you dress well, and not too well? Do you consider your air and manner of presenting yourself enough, and not too much? neither negligent nor stiff? All these things deserve a degree of care, a second-rate attention; they give an additional lustre to real merit. My Lord Bacon says that a pleasing figure is a perpetual letter of recommendation. It is certainly an agreeable forerunner of merit, and smooths the way for it."

Again, in 1749, there was the following admonition:

"Pray send for the best operator for the teeth, at Turin, where, I suppose, there is some famous one; and let him put yours in perfect order; and then take care to keep them so, afterwards, yourself. You had very good teeth, and I hope they are so still; but even those who have bad ones should keep them clean; for a dirty mouth is, in my mind, ill manners."

In 1751, the father admonished his son yet again, as follows:

"I hope you take infinite care of your teeth; the consequences of neglecting the mouth are serious not only to one's self, but to others."

In general, Chesterfield gave the following advice, which might almost be attributed to our Colonial philosopher whose sage counsels in *Poor Richard's Almanac* appeared at the same time:

"Young fellows, thinking they have so much health and time before them, are very apt to neglect or lavish both, and beggar themselves before they are aware; whereas, a prudent economy in both would make them rich indeed; and so far from breaking in upon their pleasures, would improve and almost perpetuate them. Be you wiser; and, before it is too late, manage both with care and frugality; and lay out neither, but upon good interest and security."

The parting salutation of a Chesterfield letter is deserving of more attention than it generally receives:

"Adieu, child. Take care of your health; there are no pleasures without it."—Cheesman A. Herrick in *Cosmos.*

*Devitalizing for Crowns.* In adapting shell crowns to molars or bicuspids, is it necessary to devitalize? My own opinion is that in the vast majority of cases, I would say at least ninety per cent., proper preparation can not be made without devitalizing. My opinion is based on our experience at the hospital clinic. In the majority of cases where a shell crown is to be adapted, students will ask the question: "Shall I devitalize this tooth?" My answer is: "Try it first, and if the pain becomes too excessive, then devitalize." In nearly all cases we have to resort to devitalization.—A. W. Thornton, in *Dominion Dental Journal.*

*Sloppy Amalgam.* Weigh your portions of alloy and mercury and do the first stages of the mixing by putting same into a rubber finger and mix thoroughly for one minute, then put the mass on a piece of coarse paper and complete the mixing, by drawing a heavy putty knife over the mass a number of times, which makes a very good plastic mass. Do not squeeze out the surplus mercury now, but pack into the cavity, using heavy hand pressure, with instruments which force the mercury to the surface and leave a very compact mass below the surface; surplus mercury is now scraped off with a thin instrument; repeat until the cavity is more than full and let rest for a few minutes, then restore normal occlusion by carving. Fill a large molar cavity in the laboratory, by this method, let rest for a day, then polish, and see the beautiful result.—W. O. Fellman, in *Review.*

